# Supplementary material for: Leydig Cells in Immunocastrated Polish Landrace Pig Testis: Differentiation Status and Steroid Enzyme Expression Status
Source: Int J Mol Sci. 2022 May 30;23(11):6120. doi: 10.3390/ijms23116120 (PMC9181736; doi:10.3390/ijms23116120)
Supplement: Supplementary file 1 [file ijms-23-06120-s001.zip › ijms-1712092-supplementary.pdf]

Leydig cells in immunocastrated Polish Landrace pig testis: differentiation status and steroid enzyme expression status

Piotr Pawlicki[1], Anna Galuszka[2], Laura Pardyak[1], Ryszard Tuz[3], Bartosz J. Plachno[4], Martyna Malopolska[5], Klaudia Dubniewicz[6], Ping Yang[7], Malgorzata Kotula-Balak[2], Kazimierz Tarasiuk[6]

[1]Center of Experimental and Innovative Medicine, University of Agriculture in Krakow, Redzina 1c, 30-248 Krakow, Poland
[2]Department of Animal Anatomy and Preclinical Sciences, University Centre of Veterinary Medicine JU-UA, University of Agriculture in Krakow, Mickiewicza 24/28, 30-059, Krakow, Poland
[3]Department of Genetics, Animal Breeding and Ethology, Faculty of Animal Science, University of Agriculture in Krakow, Mickiewicza 24/28, 30-059, Poland

[4]Department of Plant Cytology and Embryology, Institute of Botany, Jagiellonian University in Krakow, Gronostajowa 9, 30-387 Krakow, Poland
[5]Department of Pig Breeding, National Research Institute of Animal Production, Krakowska 1, 32-083, Balice n. Krakow, Poland
[6]Department of Infectious Diseases of Animals and Food Hygiene, University Centre of Veterinary Medicine JU-UA, University of Agriculture in Krakow, Mickiewicza 24/28, 30-059, Krakow, Poland
[7]MOE Joint International Research Laboratory of Animal Health and Food Safety, College of Veterinary Medicine, Nanjing Agricultural University, Nanjing, China

Correspondence to:
Prof. Malgorzata Kotula-Balak; e-mail: Malgorzata.Kotula-Balak@urk.edu.pl

Supplementary Tables

Table S1. Sequences of forward and reverse primers.

Gene	GenBank ID	Sequence (5' → 3', Forward primer/Reverse primer)	Product length (bp)
HSD3B1	XM_021088745.1	ATCTGCAGGAGATCCGGGTA<br>TTCAGGCACTGCTCATCCAG	127
HSD17B11	XM_021101772.1	GGATGCATAGTCCAGGAATGC<br>TGTCTGCAGTCCAGTTGCTT	127
CYP11A1	XM_021098320.1	CTACCGCCTCCTGGGAAATG	133

		CAGGCTACGTGCCATCTCAT	
CYP19A1	NM_214429	GATCCCACTGGACGAAAAGGC<br>TACTTTCTGTACAGCCAAGGAATC	117
SRD5A1	XM_003134156.6	GGTACTTGAGCCAGTACGCC<br>TCCCCTGGTTTCCTGAGGTT	142
ACTB	XM_021086047.1	TCAGCAAGCAGGAGTACGAC<br>TGCAGGTCCCGAGAGAATGA	99
GAPDH	XM_021091114.1	AAGCATGTGGGGGACTTGGA<br>AGTTAAAAGCAGCCCTGGTGA	101
B2M	NM_213978.1	TCAACCACTTTTCACACCGC<br>TCAACCACTTTTCACACCGC	130

Table S2. Primary antibodies used for Western blot and immunohistochemistry.

Antibody	Host species	Vendor	Catalog no.	Application(s)/dilution(s)
3β-HSD	Rabbit	Affinity	DF3653	IHC (1:200)<br>WB (1:500)
17β-HSD	Rabbit	Fine Biotech	FNab04023	IHC (1:100)<br>WB (1:500)
P450scc	Rabbit	Abcam	ab49129	IHC (1:200)<br>WB (1:700)
P450arom	Rabbit	Affinity	DF6884	IHC (1:100)<br>WB (1:500)
5α-reductase	Rabbit	QnD	QD1202681	IHC (1:100)<br>WB (1:500)
INSL3	Rabbit	Santa Cruz Biot.	sc-134587	IHC (1:100)

RLN	Goat	Santa Cruz Biot.	sc-20491	IHC (1:200)
PDGFRα	Rabbit	Cell Signaling Technology	#3174	WB (1:300)
β-actin	Mouse	Mouse Sigma-Aldrich	A2228	WB (1:2000)
